# The dimeric conformation of PRRSV nsp1α is important for its ability to regulate viral RNA synthesis

**DOI:** 10.1186/s13567-025-01537-5

**Published:** 2025-05-21

**Authors:** Qingyu Li, Jingbo Hu, Xue Jiao, Jing Shi, Chenxi Li, Yanhua Li

**Affiliations:** 1 College of Veterinary Medicine, Yangzhou University, Yangzhou, 225009 Jiangsu China; 2 Comparative Medicine Research Institute, Yangzhou University, Yangzhou, 225009 Jiangsu China; 3 Jiangsu Coinnovation Center for Prevention and Control of Important Animal Infectious Diseases and Zoonoses, Yangzhou, 225009 Jiangsu China

**Keywords:** PRRSV, nsp1α dimerization, viral RNA synthesis, subgenomic RNA, transcriptional regulation

## Abstract

PRRSV nsp1α, the first viral protein translated in virus-infected cells, is released from viral polyprotein 1a through autocleavage. It plays important roles in viral replication, the suppression of the host innate immune response, and the modulation of cell-mediated immune responses. Nsp1α forms a homodimer in vitro. In this study, we aimed to elucidate the functional significance of nsp1α dimerization. Using the alanine scanning strategy, we identified valine132 and proline134 as critical residues for nsp1α dimerization. Using recombinant viruses expressing an additional FLAG-nsp1α mutant (V132A or P134A), we demonstrated that both the V132A and P134A mutations disrupted nsp1α dimerization in PRRSV-infected cells. When ectopically expressed, the V132A or P134A mutation did not affect the ability of nsp1α to antagonize host type I IFN production or degrade SLA-I molecules. Introducing V132A or P134A mutations into an HP‒PRRSV replicon system significantly interfered with the expression of a Gaussia luciferase reporter and viral proteins, suggesting that nsp1α dimerization is critical for viral replication. Using PRRSV reverse genetics, a recombinant virus carrying the V132A mutation (vV132A) was successfully rescued, while the P134A mutation was lethal. Compared with the wild-type virus, vV132A significantly attenuated growth and reduced the relative expression levels of subgenomic RNAs in MARC-145 cells. In BHK-21 cells transfected with full-length cDNA clones, the P134A mutation nearly completely blocked the synthesis of specific sgRNAs at both the minus- and positive-strand levels while maintaining sgRNA6 accumulation. Thus, nsp1α dimerization is essential for viral RNA synthesis and transcriptional regulation but appears to be dispensable for both the autoproteolytic activity and immune evasion functions of PCPα. This study not only enhances our fundamental knowledge of PRRSV biology but also establishes a foundation for developing targeted antiviral strategies against PRRSV and related arteriviruses.

**Supplementary Information:**

The online version contains supplementary material available at 10.1186/s13567-025-01537-5.

## Introduction

Since its initial identification as a “mystery swine disease” in the United States in 1987, porcine reproductive and respiratory syndrome (PRRS) has remained a major global concern for more than three decades. The disease is characterized by reproductive failure in pregnant sows and respiratory distress in pigs of all ages. The etiological agent, porcine reproductive and respiratory syndrome virus (PRRSV), is classified within the family *Arteriviridae* under the order *Nidovirales*. According to the International Committee on Taxonomy of Viruses (ICTV), PRRSV is categorized into two distinct species: *Betaarterivirus suid 1* (PRRSV-1) and *Betaarterivirus suid 2* (PRRSV-2) [1]. PRRSV-2 exhibits high genetic variability and can be further divided into nine lineages on the basis of ORF5 sequence analysis [2, 3]. In China, a highly pathogenic PRRSV (HP-PRRSV) strain emerged in 2006 [4, 5], followed by the rapid dissemination of NADC30-like strains in 2013 [6, 7]. In 2017, the NADC34-like strain was first reported and gradually became predominant in China [8]. Owing to its extensive genetic variability and rapid evolutionary rate under field conditions, PRRSV remains a significant threat to global swine production, causing substantial economic losses worldwide.

The PRRSV genome consists of a single-stranded, positive-sense RNA approximately 15 kb in length, featuring a 5′ cap and a 3′ polyadenylated tail [9]. It contains at least eleven open reading frames (ORFs). ORF1a and ORF1b encode two large polyproteins (pp1a and pp1ab), which are further processed into 14 nonstructural proteins (nsps) by viral proteases, including nsp1α/β, nsp2, and nsp4. Additionally, two nonstructural proteins, nsp2TF and nsp2N, are translated through a novel -2/-1 programmed ribosomal frameshifting [10, 11]. PRRSV employs a discontinuous transcription strategy to generate a nested set of commercial subgenomic mRNAs (sg mRNAs), which encode eight structural proteins: E, GP2a, GP3, GP4, GP5, ORF5a, M, and N [12, 13]. These nsps are believed to assemble into a membrane-associated replication and transcription complex (RTC), which plays a pivotal role in regulating PRRSV RNA synthesis [12, 13]. For example, PRRSV nsp12 is required for the synthesis of subgenomic RNAs [14]. However, the precise functions of nonstructural proteins in PRRSV RNA synthesis remain largely elusive.

PRRSV nsp1α comprises an N-terminal zinc finger domain, a central papain-like cysteine protease (PCP) domain, and a C-terminal extension region [15]. It has been shown to self-interact, forming a homodimer in solution, as demonstrated by co-immunoprecipitation assay in an overexpression system [16]. As a component of the RTC, nsp1α plays an important role in PRRSV RNA synthesis. During PRRSV infection, the PCPα domain of nsp1α specifically modulates subgenomic RNA (sgRNA) synthesis without affecting genomic RNA (gRNA) production [17]. In *Arteriviruses*, the zinc finger domain of nsp1 is presumed to act as a discontinuous transcription factor [18, 19]. In equine arteritis virus (EAV), nsp1α remains uncleaved from nsp1 and localizes to the nucleus [20]. EAV nsp1 is responsible for sgRNA synthesis instead of gRNA synthesis, modulating the synthesis of minus-strand subgenomic RNAs to control the relative abundance of viral mRNAs [21]. Despite these findings, the precise molecular mechanisms underlying PRRSV nsp1α-mediated modulation of viral RNA synthesis, including the structural determinants governing its functional activity, remain incompletely understood and require comprehensive investigation.

In addition to its role in viral RNA synthesis, PRRSV nsp1α is involved in virus‒host interactions, particularly in the suppression of host immune responses. Nsp1α employs multiple mechanisms to suppress NF-κB activation, such as reducing the LUBAC-dependent linear ubiquitination of NEMO and hijacking upregulated host ASB8 to degrade IKKβ [22–26]. Additionally, nsp1α inhibits type I IFN promoter activity through degrading CREB-binding protein (CBP) in the nucleus, with its zinc finger domain playing a critical role in this process [27, 28]. During PRRSV infection, nsp1α targets swine leukocyte antigen class I (SLA-I) for degradation through the proteasome pathway, thereby downregulating SLA-I-mediated cellular immunity [29, 30]. Furthermore, nsp1α promotes the secretion of sCD83, which impairs the ability of monocyte-derived dendritic cells (MoDCs) to stimulate T-cell proliferation [31].

To date, the key residues involved in nsp1α dimerization have not been identified, and no investigations have been conducted to dissect the structure‒function relationship. To elucidate the structural and functional significance of the dimeric conformation of nsp1α, we generated and characterized dimerization-deficient mutants and systematically analysed their impact on viral RNA synthesis and virus‒host interactions. Through comprehensive mutagenesis analysis, we identified valine 132 and proline 134 as critical residues essential for nsp1α dimerization. Our study revealed that while nsp1α dimerization is dispensable for the immunosuppressive function of nsp1α, it plays a crucial regulatory role in subgenomic RNA (sgRNA) synthesis by modulating the relative accumulation levels of sgRNA species.

## Materials and methods

### Cells and viruses

HEK-293T cells and BHK-21 cells purchased from ATCC were maintained in Dulbecco’s modified Eagle’s medium (DMEM) (Cytiva, USA) supplemented with 10% fetal bovine serum (Vazyme Biotech, China) and 1% penicillin‒streptomycin (Thermo Fisher Scientific, USA). MARC-145 cells from the ATCC were cultivated in modified Eagle’s medium (MEM; Cytiva, USA) supplemented with 10% FBS (Sigma‒Aldrich, USA) and 1% penicillin‒streptomycin (Thermo Fisher Scientific). All the cells were cultured at 37 °C with 5% CO_2_ in a humidified incubator (Thermo Fisher Scientific). The highly pathogenic PRRSV (HP-PRRSV) strain TA-12 (accession no. HQ416720.1) and the reporter virus TA-EGFP containing an EGFP gene in the nsp2-coding region were used in this study.

### Antibodies

A rabbit polyclonal antibody (pAb) against PRRSV nsp1α was kindly provided by Dr Changjiang Weng from Harbin Veterinary Research Institute, CAAS. A monoclonal antibody against PRRSV N generated in our laboratory was used for western blot analysis and immunofluorescence assays. PAbs against PRRSV M and GP5 were purchased from GeneTex (Southern California, USA). A rabbit polyclonal antibody against PRRSV nsp2 was generated by GenScript (Nanjing, China). The following antibodies from various sources were also used in this study: an anti-β-tubulin pAb (Bioworld, USA), an anti-β-actin mAb (Proteintech, China), an anti-GAPDH pAb (Bioworld), and mAbs against FLAG-tag (MBL, Japan) and HA-tag (BioLegend, USA).

### Plasmids

**Figure 1:**
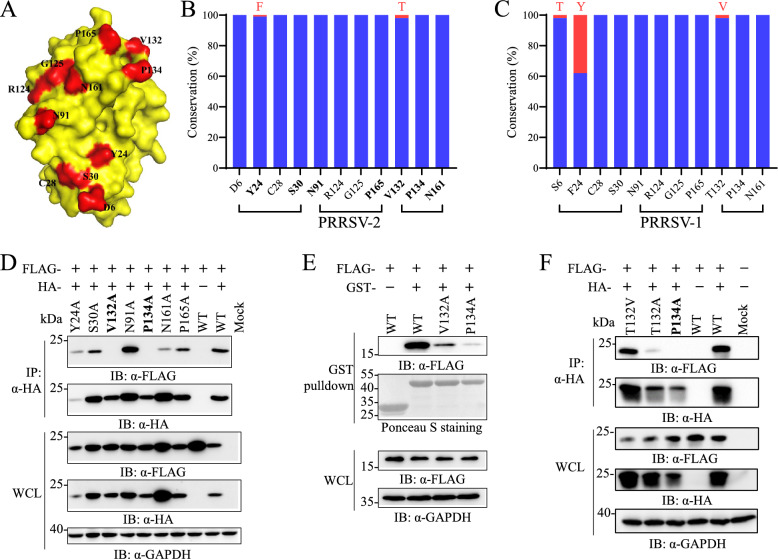
**Mapping the key residues involved in PRRSV nsp1α dimerization.** **A** The residues that potentially mediate PRRSV nsp1α dimerization are highlighted in red, while the self-interaction surface is highlighted in yellow. This picture was adapted from Sun et al. [15]. **B**, **C** Conservation analysis of the residues that potentially mediate nsp1α dimerization in PRRSV-2 and PRRSV-1 strains. **D** Co-IP analysis of the self-interaction of the nsp1α mutants in PRRSV-2. A image of nsp1α mutants was created by the alanine scanning strategy. For each mutant, plasmids expressing N-terminal FLAG-tagged or HA-tagged nsp1α were constructed. HEK293T cells were transfected with plasmids expressing two versions of nsp1α or its mutants. At 36 hpt, the cell lysates were harvested for immunoprecipitation with anti-HA magnetic beads (Selleck), followed by western blot analysis with antibodies against the HA tag and FLAG tag. GAPDH was detected as a loading control. **E** The direct self-interaction ability of nsp1α was experimentally validated in vitro through GST pulldown assays. Specifically, GST-conjugated magnetic beads were immobilized with GST alone (negative control), GST-nsp1α, or its dimerization-deficient variants (GST-V132A and GST-P134A). These bait proteins were then incubated with cell lysates containing corresponding prey proteins, including Flag-tagged nsp1α and its mutant derivatives (FLAG-V132A and FLAG-P134A). The pulldown of FLAG-tagged nsp1α or its mutants was detected by western blotting with an anti-FLAG antibody, while equal inputs of GST alone, GST-nsp1α, or its mutants were verified by Ponceau S staining. **F** Co-IP analysis of the self-interaction of the nsp1α mutants in PRRSV-1. The threonine 132 and proline 134 residues of nsp1α were targeted for mutagenesis analysis. For each mutant, plasmids expressing N-terminal FLAG-tagged or HA-tagged nsp1α were constructed. HEK293T cells were transfected with plasmids expressing two versions of nsp1α or its mutants. At 36 hpt, the cell lysates were harvested for immunoprecipitation with anti-HA magnetic beads (Selleck), followed by western blot analysis with antibodies against the HA tag and FLAG tag. GAPDH was detected as a loading control.

Plasmids expressing nsp1α from the HP-PRRSV TA-12 strain and PRRSV-1 SHE strain (accession no. GQ461593.1) with an N-terminal FLAG tag or an HA tag were generated by inserting their coding regions into the pCAGGS vector. The coding sequences carrying mutations at the selected residues (Figures 1D and F) were PCR amplified using primers listed in Table 1 and inserted into pCAGGS using a ClonExpress Ultra One Step Cloning Kit (Vazyme Biotech). For prokaryotic expression, the coding regions of HP-PRRSV nsp1α and its mutants (V132A or P134A) were cloned and inserted into the pET-HisGST vector to express GST-fusion proteins in *E. coli*. The coding regions of SLA-I-HC and SLA-I-β2 m were amplified by RT‒PCR with the primers listed in Table 1 and subsequently cloned and inserted into the pcDNA-EGFP-P2A vector. A replicon of the TA-12 strain, pCMV-TA-EGFP-rep, was created by replacing the coding regions of envelope proteins with Gaussia luciferase, and an EGFP was inserted in the nsp2-coding region. With the primers listed in Table 1, the V132A and P134A mutations were introduced into the replicon to generate mutants. All the constructs were verified by DNA sequencing.

**Table 1:** **The primers used for plasmid construction**

Name	Sequence (5′-3′)
FLAG-1α-F	ATTCGAGCTCGCCACCATGGATTACAAGGATGACGACGATAAGTCCGGGATACTTGATCGG
HA-1α-F	TTCGAGCTCGCCACCATGTACCCATACGATGTTCCAGATTACGCTTCCGGGATACTTGATCGG
1α-R	TAGCTCGAGCTACATAGCACACTCAAAAGG
SacI-F	CATTTTGGCAAAGAATTCG
XhoI-R	GGGAAAAAGATCTGCTAGC
1α-Y24A-F	GCCAGGTCGCCTGCACACGAT
1α-Y24A-R	TCGTGTGCAGCAGACCTGGC
1α-S30A-F	CACGATGTCTCGCTGCACGGTCTCT
1α-S30A-R	ACCGTGCACGGAGACATCGTG
1α-N91A-F	ACTAGTGGAGCCCTGAACTTTCAACAAAG
1α-N91A-R	AAGTTCAGGCGTCCACTAGTCATTCG
1α-V132A-F	CCCATTGCCGGGCCCGT
1α-V132A-R	CGGGCCCGCCAATGGGGTA
1α-P134A-F	ATTGTCGGGGCCGTCCCTG
1α-P134A-R	CCAGGGACGGCCCCGACAAT
1α-N161A-F	CATGTGTTAACCGCTTTGCCGCTCC
1α-N161A-R	GGAGCGGCAAAGCGGTTAACACATG
1α-P165A-F	GCCGCTCGCGCAGAGGC
1α-P165A-R	GGCCTCTGCGCGAGCGGCAAATT
nsp1-C76S-F	GCCGGGGCCTCCTGGCTTTCTGC
nsp1-C76S-R	GATCGCAGAAAGCCAGGAGGCCCC
nsp1-XhoI-R	TAGCTCGAGCTAACCGTACCACTTATA
SHE-1α-F1	ATCATTTTGGCAAAGAATTCGAGCTCGCCACCATGTACCCATACGATGTTCCAGATT
SHE-1α-F2	CCATACGATGTTCCAGATTACGCTATGTCTGGGACGTTCTCCCGGTGCATGTG
SHE-1α-R	GGAAAAAGATCTGCTAGCTCGAGCTAATGAGCCTCCTCAAATGG
SHE-T132A-F	CCGATCGCGGGGCCCGTGCCCGG
SHE-T132A-R	CGGGCACGGGCCCCGCGATCG
SHE-T132V-F	CTGGTACCCGATCGTGGGGCCCGTG
SHE-T132V-R	CACGGGCCCCACGATCGGGTA
SHE-P134A-F	ATCACGGGGGCCGTGCCCGGGA
SHE-P134A-R	GGCACGGCCCCCGTGATCGGG
MluI-F	AGGTCTATATAAGCAGAGCTACGCGTTA
1α-V132A-R1	ACGGGCCCGCCAATGGGGTAC
1α-V132A-F1	TACCCCATTGCCGGGCCCGT
1α-P134A-F1	CCATTGTCGGGGCCGTCCCTGGG
1α-P134A-R1	CCAGGGACGGCCCCGACAATG
AflII-R	GGGGCAGGAAGGCATAGGTG
SLA-1-F	CCTGAATTCATGGGGCCTGGAGCCCT
SLA-1-R	ATCCTCGAGCACTCTAGGACCCTTGGTAAG
B2M-F	CCTGAATTCATGGCTCCCCTCGTGGC
B2M-R	ATCCTCGAGGTGGTCTCGATCCCACTTA

### PRRSV reverse genetics

Using the TA-12 infectious cDNA clone [32], three full-length cDNA clones were generated by inserting an expression cassette that is the coding region of FLAG-nsp1α or its mutants (V132A or P134A) followed by a TRS6 sequence. To create a reporter virus, a full-length cDNA clone carrying the EGFP gene in the highly variable region of nsp2 was created by NEBuilder® HiFi DNA Assembly Master Mix (NEB, USA) and designated TA-EGFP. Similarly, V132A and P134A were introduced into TA-EGFP to create cDNA clones containing the corresponding nsp1α mutants.

The recombinant viruses were rescued by DNA transfection of BHK-21 cells and inoculation of MARC-145 cells. Briefly, BHK-21 cells at 70–80% confluency were transfected with 2 μg of full-length cDNA clone using Lipofectamine™ 3000 transfection reagent (Invitrogen, USA) according to the manufacturer’s instructions. At 48 h post-transfection (hpt), the culture supernatant was harvested to infect MARC-145 cells. The success of viral recovery was monitored daily by the cytopathic effect (CPE) or EGFP reporter expression. The recombinant viruses were serially passaged in MARC-145 cells. The correct introduction of mutations or insertions in the recombinant viruses was verified by sequencing the corresponding viral genomic regions.

### Viral growth curve

A viral growth curve was generated to characterize the recombinant viruses, wild-type virus and vV132A mutant. Briefly, MARC-145 cells seeded in 24-well plates were infected with passage 3 viruses at a multiplicity of infection (MOI) of 0.01. At 2 h post-infection (hpi), the cells were washed three times with 1 × phosphate-buffered saline (PBS) and maintained in MEM supplemented with 2% FBS. The culture supernatants were harvested at 12, 24, 36, 48, 60, and 72 hpi. The viral progeny in the supernatants were titrated and calculated as the 50% tissue culture infective dose per millilitre (TCID_50_/mL) using the Reed‒Muench method.

### Plaque assay

A plaque assay was performed to determine the plaque morphology of the recombinant viruses. Briefly, cells grown in 6-well plates were infected with serially diluted WT or vV132A mutant strains. At 2 hpi, the cell monolayers were washed with 1 × PBS and overlaid with a mixture of 1% UltraPure™ Low Melting Point Agarose (Invitrogen) and MEM supplemented with 2% FBS. At approximately 4 days post infection (dpi), the cell monolayers were fixed with 4% paraformaldehyde for several hours, followed by staining with 0.1% crystal violet for 10 min to visualize the plaques.

### Immunofluorescence assay (IFA)

MARC-145 cells grown in glass bottom dishes or cell culture plates were inoculated with recombinant viruses. At 24 hpi or 36 hpi, the cells were fixed with ice-cold methanol for 20 min. After being blocked with 2% bovine serum albumin (BSA) in 1 × PBS at room temperature for 30 min, the cell monolayers were incubated with primary antibodies diluted with 2% BSA at 4 °C overnight. After five washes with PBS, the cells were incubated with secondary antibodies (Jackson ImmunoResearch, Inc., USA) at 37 °C for 1 h. The nuclei were stained with 4′,6-diamidino-2-phenylindole (DAPI; Solarbio Life Sciences, China) for 5 min at room temperature. The cells were examined under an IX73 epifluorescence microscope (Olympus, Japan), and images were captured.

### Dual-luciferase reporter assay

The p125-luc reporter plasmid was used to evaluate the inhibitory effects of nsp1α and its mutants on IFN-β production. HEK-293T cells grown in 24-well plates were transfected with 1 μg of a plasmid expressing PRRSV-2 nsp1α or its mutants, 0.1 μg of the reporter plasmid p125-luc, 0.01 μg of pRL-SV40, or a plasmid expressing the N-terminal CARD domain of RIG-I or an empty vector using the Lipofectamine 2000 transfection reagent (Invitrogen). At 24 hpt, the cell lysates were harvested for a luciferase assay using a Dual-Luciferase Reporter Assay Kit (Vazyme Biotech). The transcriptional activity of the IFN-β promoter was quantified by dual-luciferase reporter assay, with the results expressed as relative luciferase activity calculated by normalizing firefly luciferase activity to Renilla luciferase activity.

### HP-PRRSV replicon assay

HEK-293T or BHK-21 cells seeded in 24-well plates were transfected with 1 μg of pCMV-TA-EGFP-rep or its mutants (V132A and P134A). At 24 hpt, the culture supernatants were harvested for the Gaussia luciferase assay. Briefly, 20 µL of culture supernatant was mixed with 50 μL of coelenterazine h (20 μM) in a white plate, and photon counts were acquired for 10 s with a plate reader (Shanpu, China).

### Western blot analysis

Western blot analysis was performed to evaluate protein expression. Briefly, cell lysates were prepared using IP lysis buffer (Biosharp, China). The cell debris was removed by centrifugation at 12 000 × *g* at 4 °C for 10 min. The lysates were mixed with 5 × SDS‒PAGE loading buffer containing β-mercaptoethanol and boiled at 95 °C for 10 min. After separation by sodium dodecyl sulfate‒polyacrylamide gel electrophoresis (SDS‒PAGE), the proteins were transferred onto a nitrocellulose membrane (Millipore, USA). The membranes were blocked with 5% skim milk in 1 × PBS at room temperature for 1 h, followed by incubation with the appropriate primary antibodies at the recommended dilutions at 4 °C overnight. After extensive washes with 1 × PBS supplemented with 0.05% Tween-20, the membrane was incubated with HRP-conjugated goat anti-rabbit IgG or goat anti-mouse IgG secondary antibodies at the recommended dilution for 1 h at room temperature. The target protein bands were visualized with an enhanced chemiluminescence (ECL) substrate (Biosharp, China) using a Tanon 5200 multi-imaging system.

### Co-immunoprecipitation (Co-IP) assay

HEK-293T cells transfected with the indicated plasmids were lysed with IP lysis buffer (Biosharp, China) supplemented with protease inhibitors and 1 mM phenylmethylsulfonyl fluoride (PMSF; Beyotime, China). The cell debris was removed by centrifugation at 12 000 *g*, 4 ℃, for 15 min. Immunoprecipitation was conducted using the Anti-HA magnetic beads (Selleckchem, USA) according to the manufacturer’s instructions. The clarified lysates were mixed with the beads overnight at 4 ℃. The beads were collected with a magnetic separation stand and washed five times with 1 × PBS containing 0.05% Tween-20 (PBST). The immunocomplexes on the beads were eluted using the 5 × SDS-PAGE loading buffer containing β-mercaptoethanol, followed by boiling at 95 ℃ for 5 min. Western blotting was performed to analyse the protein complexes using antibodies against the HA tag and FLAG tag.

### GST pulldown assay

*E. coli* BL21 cells containing the plasmids pET-HisGST-nsp1α, pET-HisGST-nsp1α-V132A, pET-HisGST-nsp1α-P134A, or pET-HisGST were cultured in 2 × YT medium at 37 ℃. Protein expression was induced with 1 mM isopropyl β-D-1-thiogalactopyranoside (IPTG; Solarbio Life Sciences, China) for 24 h at 22 ℃ when the OD600 of the *E. coli* cells reached 0.6. The cultures were harvested by centrifugation at 4000 rpm for 15 min and resuspended in 1 × PBS (pH 7.4) supplemented with protease inhibitors, followed by sonication. The cell debris was removed by centrifugation for 30 min at 12 000 *g*, 4 ℃. The lysates were incubated with GST-conjugated magnetic beads at 4 ℃ for 2 h. Next, after being washed 5 times with wash buffer (50 mM Tris–HCl pH 7.5; 150 mM NaCl; 1 mM EDTA), the beads were used for the GST pulldown assay using the GST IP Kit (ApalifeBio, China) according to the manufacturer’s instructions. HEK-293T cells expressing FLAG-nsp1α or its mutants were harvested with lysis buffer (50 mM Tris–HCl pH 7.5; 150 mM NaCl; 1% Triton-100; 1 mM EDTA) supplemented with protease inhibitors at 30 hpt. To assess the interaction between GST-nsp1α and FLAG-nsp1α, the beads were mixed with HEK-293T cell lysates expressing the corresponding proteins and incubated in a rotator at 4 °C overnight. Following 5 consecutive washes with wash buffer, the protein‒protein complexes were eluted using 5 × SDS‒PAGE loading buffer containing β-mercaptoethanol and boiled at 95 °C for 5 min. The samples were separated on SDS‒PAGE gels and transferred onto NC membranes. The input of the GST-fusion proteins was confirmed by the Ponceau S staining. The membranes were probed with specific primary antibodies followed by appropriate secondary antibodies for target detection.

### Quantitative reverse transcription PCR (RT‒qPCR)

BHK-21 cells transfected with full-length cDNA clones of the TA-12 strain were harvested at 24 hpt using the TRIzol reagent (Vazyme Biotech) and subjected to total cellular RNA extraction according to the manufacturer’s instructions. Similarly, MARC-145 cells infected with vTA-EGFP (WT) or TA-EGFP-V132A (vV132A) at an MOI of 3 were harvested at 9 hpi for total cellular RNA extraction. To quantify viral RNA species, including both genomic and subgenomic RNAs of positive and negative polarities, reverse transcription‒quantitative PCR (RT‒qPCR) was performed. Briefly, 1 µg of total RNA was treated with DNase I to remove genomic DNA contamination. First-strand cDNA was synthesized using the HiScript III 1st Strand cDNA Synthesis Kit (Vazyme Biotech) according to the manufacturer’s instructions. For the detection of negative-sense viral RNAs, the primer SGen-F was used for cDNA synthesis. For positive-sense genomic and subgenomic RNAs, the primers nsp1β-R and SGen-R, respectively, were used. In addition, the expression of the housekeeping gene β-actin was also quantified. Real-time PCR amplification was carried out using the ChamQ Universal SYBR qPCR Master Mix (Vazyme Biotech) with the generated cDNA templates and specific primers listed in Table 2. The reaction program was set as follows: 95 °C for 30 s, followed by 40 cycles at 95 °C for 10 s and 60 °C for 20 s. The relative abundance of each viral RNA species was normalized to the expression of β-actin mRNA using the 2^−ΔΔCt^ method.

**Table 2:** **The primers used for RT‒qPCR quantification of PRRSV RNA**.

Name	Sequence (5′-3′)
gRNA-F	CTCCACCCCTTTAACCATGTC
gRNA-R	AATGCACGTGGCAACGTCCAC
sgRNA2-F	CTCTCCACCCCTTKAACCAACTTT
sgRNA2-R	CGGAGCAAACCAGTCTGATGC
sgRNA3-F	CTCCACCCCTKTAACCATAGTG
sgRNA3-R	CCCCTAACCAGCGGAAACCA
sgRNA4-F	CTCCACCCCTTTMACCTGGAA
sgRNA4-R	TGAGGACTTTTGCGAATCGTCG
sgRNA5-F	CTCCACCCCTTTARCCTGTCT
sgRNA5-R	CCAATCTGTGCCATTCAGCTC
sgRNA6-F	CTCCACCCCTTTAACCAGAGTTT
sgRNA6-R	GATCAAAAGGTGCAGAAGCCC
sgRNA7-F	CTCCACCCCTWTAACCACGCAT
sgRNA7-R	ACCCAGCATTTGGCACAGCT
nsp1β-R	ACCGTACCACTTATGACTGC
SGen-R	TTTTTTTTTTTAATTACGGCCG
SGen-F	TGTGACAGCTCTCTTCAGGG
actb-qF	CCCTGGAGAAGAGCTACGAG
actb-qR (BHK-21)	GGAAGGAAGGCTGGAAGAGT
actb-qR1 (MARC-145)	CAGGAAGGAAGGTTGGAAGAG

### Statistical analysis

Statistical analyses were performed with GraphPad Prism software 9.5.1. The data are presented as the means ± SD from at least three replicates.

## Results

### Identification of critical residues for PRRSV nsp1α dimerization

PRRSV nsp1α exists as a homodimer in solution, and eleven residues potentially mediating this dimer conformation were identified on the basis of its crystal structure (Figure 1A) [15]. Initially, comparative sequence analysis with all available PRRSV nsp1α sequences in the GenBank database revealed that these residues are highly conserved across the PRRSV-2 and PRRSV-1 strains, with only minor variations observed at tyrosine 6, phenylalanine 24, and valine 132 (Figures 1B and C). These residues form three patches on the protein surface. For further mutagenesis studies in PRRSV-2, we selected seven residues (positions 24, 30, 91, 132, 134, 161, and 165) that collectively cover these patches. An alanine screening strategy was employed to construct nsp1α mutants. For each alanine substitution, two plasmids expressing FLAG-tagged or HA-tagged nsp1α mutants were generated by site-directed mutagenesis. A Co-IP assay using HA-antibody-conjugated magnetic beads was carried out to verify the involvement of these residues in nsp1α dimerization, followed by western blot analysis to detect immunoprecipitated nsp1α proteins. The results revealed that among all the mutants, only the V132A and P134A mutations rendered the dimerization of nsp1α undetectable, suggesting that V132 and P134 play essential roles in nsp1α self-interaction (Figure 1D). Furthermore, to examine the direct self-interaction of nsp1α mutants, we expressed nsp1α and its mutants (nsp1α-V132A and nsp1α-P134A) as GST fusion proteins in *E. coli*. A GST pulldown assay was conducted using GST magnetic beads to examine the interaction between the recombinant GST-nsp1α protein expressed in *E. coli* and the corresponding FLAG-tagged protein ectopically expressed in HEK-293T cells. The results revealed that the V132A and P134A mutations significantly reduced nsp1α self-interaction, with the P134A mutation having a more pronounced effect (Figure 1E).

The involvement of the corresponding residues in PRRSV-1 nsp1α at positions 132 and 134 was also examined by Co-IP. As shown in Figure 1F, nsp1α dimerization was markedly reduced by the T132A mutation but not by the tyrosine substitution at the same residue, which is the corresponding amino acid in PRRSV-2. Consistent with the findings in the PRRSV-2 strain, the P134A mutation completely blocked nsp1α dimerization in the PRRSV-1 strain SHE. Overall, both the V132 and P134 residues play critical roles in PRRSV nsp1α dimerization, with P134 being particularly essential for this dimeric conformation.

### PRRSV nsp1α forms a dimer in infected cells

**Figure 2:**
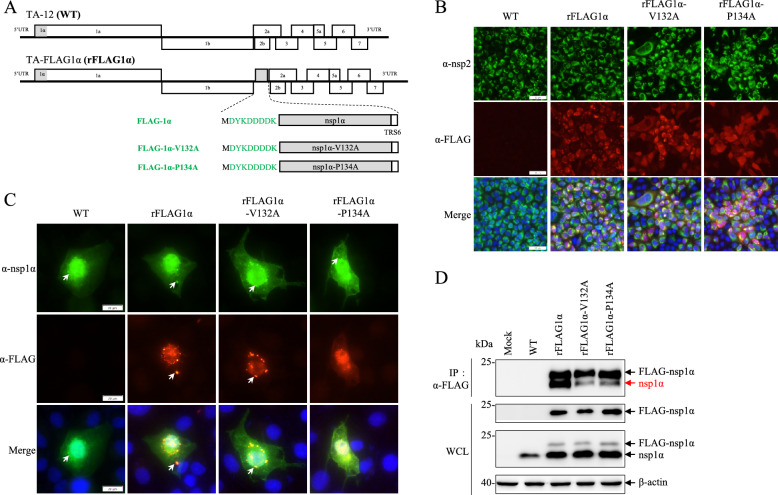
**Nsp1α can form a homodimer during PRRSV infection.** **A** Schematic diagrams of the genomic structures of the PRRSV TA-12 strain and its derivatives expressing additional nsp1α. As described previously [39], the expression cassette for FLAG-tagged nsp1α or its mutant derivatives (FLAG-V132A and FLAG-P134A) was inserted between ORF1b and ORF2a. **B** IFA detection of nsp2 and nsp1α in MARC-145 cells infected with PRRSV. MARC-145 cells were infected with WT TA-12 or its mutants expressing FLAG-tagged nsp1α or its mutant derivatives. At 48 hpi, the cell monolayers were fixed and stained for nsp2 and FLAG-tagged nsp1α using specific antibodies. Nuclei were counterstained with DAPI. The scale bar is 50 μm. **C** Colocalization of nsp1α and FLAG-tagged nsp1α. MARC-145 cells were infected with WT TA-12 or its mutants at an MOI of 0.1. At 24 hpi, the cells were fixed and stained for nsp1α and FLAG-tagged nsp1α using rabbit polyclonal antibody against nsp1α and a mouse monoclonal antibody against the FLAG tag, respectively. Nuclei were counterstained with DAPI. The scale bar is 20 μm. **D** Co-IP analysis of the interaction between nsp1α and FLAG-tagged nsp1α or its mutant derivatives (FLAG-V132A and FLAG-P134A). MARC-145 cells were infected with WT TA-12 or its mutants at an MOI of 0.1. At 24 hpi, the cell lysates were harvested for Co-IP analysis using anti-FLAG magnetic beads. The expression of nsp1α and FLAG-tagged nsp1α in whole-cell lysates (WCL) immunoprecipitated with anti-FLAG magnetic beads was analysed by western blotting using rabbit polyclonal antibody against nsp1α and a mouse monoclonal antibody against the FLAG tag, and β-actin was detected as a loading control.

To further verify the dimeric conformation of nsp1α during PRRSV infection, recombinant viruses expressing an additional nsp1α or its mutants (V132A or P134A) with a FLAG tag were constructed by the insertion of an expression cassette between ORF1b and ORF2a, as illustrated in Figure 2A. As expected, the expression of additional nsp1α was detected in cells infected with all three recombinant viruses but not in those infected with the WT virus via IFA with a FLAG-tag monoclonal antibody (Figure 2B). The correct insertion of the expression cassette in the recombinant viruses was further verified by sequencing. We subsequently investigated the subcellular localization of nsp1α and FLAG-tagged nsp1α or the mutants by confocal microscopy analysis. Consistent with previous studies [16, 29], nsp1α exhibited a dual localization pattern, being distributed in both the nuclear and the cytoplasmic compartments, with prominent perinuclear focus formation observed (Figure 2C). A robust co-localization pattern was detected at perinuclear foci between nsp1α and both FLAG-nsp1α and its V132A mutant variant, whereas the FLAG-P134A mutant exhibited significantly reduced accumulation in these perinuclear structures. These findings were verified by Co-IP assays using FLAG-tag magnetic beads. The results indicated that the V132A and P134A mutations strongly inhibited the formation of the nsp1α dimer in MARC-145 cells infected with PRRSV (Figure 2D). Collectively, these findings demonstrate that nsp1α adopts a dimeric conformation during PRRSV infection and that the V132 and P134 residues play critical roles in this molecular interaction.

### The dimeric conformation is not important for the proteinase and immune antagonistic activities of PRRSV nsp1α

**Figure 3:**
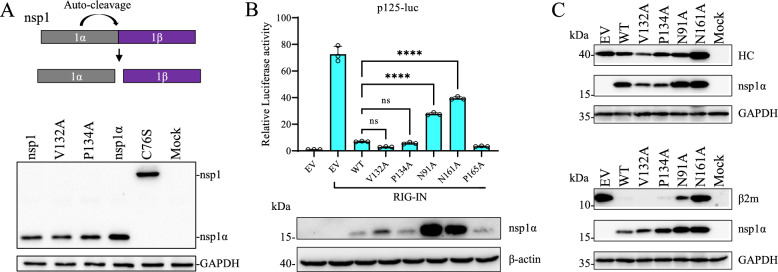
**The dimeric conformation of nsp1α has no effect on its protease activity or immune antagonistic function.** **A** The impact of nsp1α mutation on PCPα activity. The top panel shows a schematic representation of the autoproteolytic release of nsp1α from nsp1. HEK293T cells were transfected with plasmids expressing HA-nsp1α, HA-nsp1α-C76S, HA-nsp1, or nsp1 mutants (V132A and P134A). At 24 hpt, the cell lysates were harvested for western blot analysis. PCPα activity was evaluated on the basis of the release of HA-nsp1α, which was detected by an antibody against the HA tag. GAPDH was detected as a loading control. **B** The inhibitory effect of nsp1α or its mutants on the IFN-β promoter. HEK-293T cells were co-transfected with p125-Luc, pRL-SV40, a plasmid expressing nsp1α or its mutants, or a plasmid expressing RIG-IN or an empty vector (EV). At 24 hpt, the cell lysates were harvested for the dual-luciferase reporter assay. The data represent mean ± SD of three biological replicates, and the experiments were repeated three times. Statistical analysis was performed by two-tailed unpaired Student’s *t* test (****p < 0.0001; ns, no significant difference). In the bottom panel, the expression of nsp1α and its mutants was evaluated by western blot analysis using an anti-HA antibody, and GAPDH was detected as a loading control. C. The impact of mutations on nsp1α function in the degradation of SLA-I molecules. HEK293T cells were co-transfected with a plasmid expressing EGFP-SLA-I-HC (top panel) or EGFP-β2m (bottom panel) and a plasmid expressing HA-nsp1α or its mutants. At 24 hpt, the cell lysates were harvested for western blot analysis using antibodies against the HA tag, FLAG tag, or GAPDH.

PRRSV nsp1α is self-cleaved from nsp1, and its PCPα proteinase activity is responsible for this process. To test whether the dimeric conformation is critical for its proteinase activity, the V132A and P134A mutations were introduced into the nsp1-expressing construct. Western blot analysis revealed that these mutations did not affect the release of nsp1α, and the proteinase was inactivated by the C76S mutation (Figure 3A).

PRRSV nsp1α employs multiple strategies to evade host immune responses to benefit viral replication, including the suppression of innate immunity [22–25, 27] and cell-mediated immunity [29]. The effect of nsp1α dimerization on immune antagonistic activity was evaluated using a panel of nsp1α mutants. The p125-luc reporter assay revealed that the inhibitory effect of nsp1α on the activity of the IFN-β promoter was reduced by the N91A and N161A mutations but not by the V132A and P134A mutations (Figure 3B). When nsp1α or its mutants were co-expressed with SLA-I or β2m in HEK-293 T cells, the degradation of SLA-I-HC and SLA-I-β2m was determined by western blot analysis. In line with previous reports [29, 30], nsp1α targeted SLA-I for degradation, and its mutants (N91A and N161A) lost this activity (Figure 3C). The V132A and P134A mutants demonstrated comparable activity to that of wild-type (WT) nsp1α in degrading SLA-I, indicating that the dimeric conformation of nsp1α is not essential for its suppressive effect on SLA-I expression.

### The dimeric conformation of nsp1α is important for PRRSV replication

**Figure 4:**
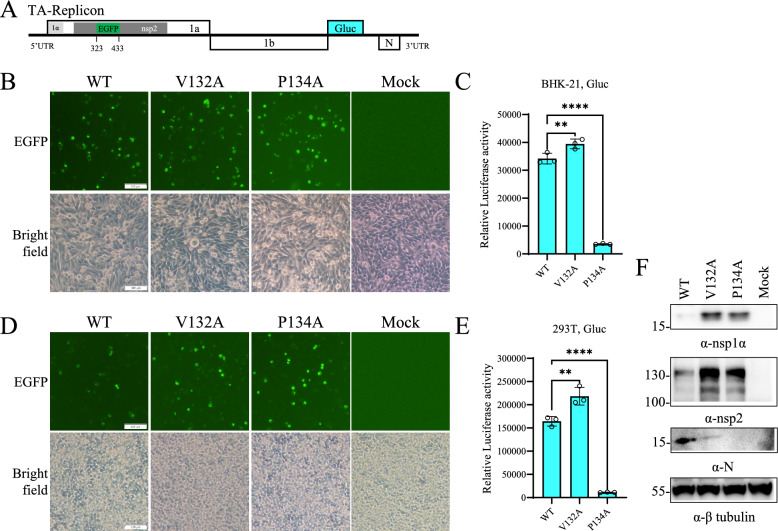
**Both the V132 and P134 residues of nsp1α are important for PRRSV replication.** **A** A replicon of the PRRSV TA-12 strain expressing EGFP and Gluc. **B–E** The impact of the V132A and P134A mutations on PRRSV replication was evaluated with the replicon system. BHK-21 cells (**B**, **C**) or HEK-293T cells (**D**–**E**) were transfected with the WT replicon or its mutants (V132A or P134A). At 24 hpt, pictures of green fluorescence and bright-field images were captured (**B** and **D**). The scale bar is 100 μm. Culture supernatants were collected for Gaussia luciferase (Gluc) activity quantification (**C** and **E**). The data are shown as the mean ± SD of three biological replicates. Statistical analysis was performed by two-tailed unpaired Student’s *t* test (**p < 0.01; ****p < 0.0001). **F** The expression of PRRSV proteins in HEK-293T cells transfected with replicons. Western blot analysis was performed to detect nsp1α, nsp2, and N using indicated antibodies. β-tubulin was detected as a loading control.

In a previous study, the PCPα activity of PRRSV nsp1α was demonstrated to be important for subgenomic mRNA synthesis but not for genomic RNA synthesis [17]. However, it is unknown whether the dimeric conformation is associated with the function of nsp1α in viral RNA synthesis. We established a replicon of the PRRSV TA-12 strain to evaluate the effects of the V132A and P134A mutations of nsp1α on PRRSV replication. As depicted in Figure 4A, EGFP was inserted in the highly variable region of the nsp2-coding region, while the coding regions of envelope proteins were replaced with a Gluc gene. The V132A and P134A mutations were introduced into nsp1α of the replicon to generate mutants. With this replicon system, PRRSV replication can be evaluated on the basis of Gluc activity in culture supernatants. The P134A mutation significantly attenuated PRRSV replication, as evidenced by a significant decrease in the Gluc activity in both BHK-21 (Figures 4B and C) and HEK-293T (Figures 4D and E) cells. In contrast, the V132A mutation increased Gluc activity (Figures 4B–E). The expression levels of nsp1α, nsp2, and N in HEK-293T cells by the replicons were examined through western blot analysis. Both the V132A and P134A mutations upregulated the expression of nonstructural proteins but downregulated N protein expression (Figure 4F). Notably, the N protein was undetectable in cells transfected with the P134A mutant. The opposite expression trends between Gluc and N in the V132A mutant suggested that the V132 residue may differentially modulate the synthesis of subgenomic RNAs. These findings reveal that residues V132 and P134 of nsp1α are important for PRRSV replication, suggesting that nsp1α dimerization is involved in PRRSV replication.

### Recovery and characterization of recombinant viruses

**Figure 5:**
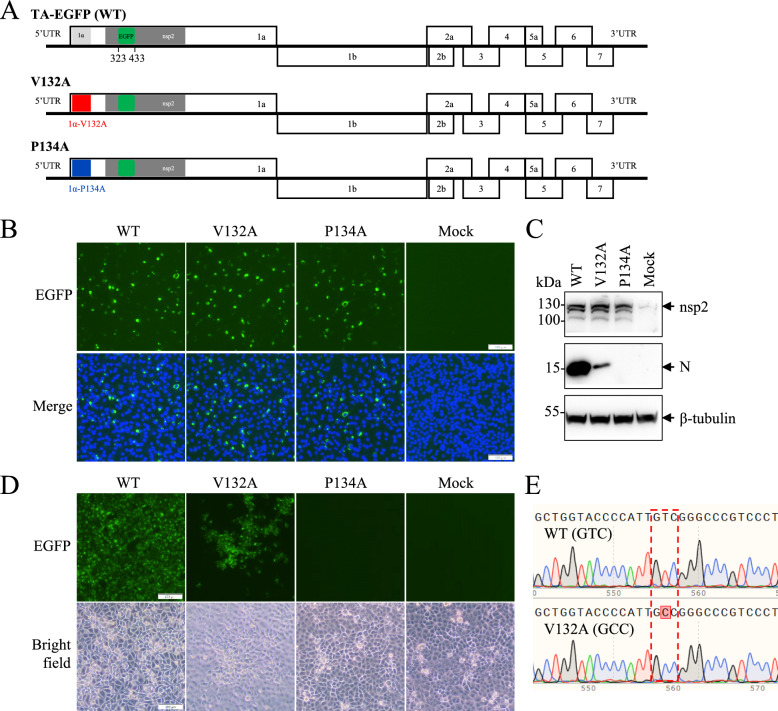
**Recovery of the recombinant PRRSV using a reverse genetics system.** **A** Genomic structures of the TA-EGFP (WT) virus and its mutants containing V132A or P134A in nsp1α. In the WT virus, an EGFP gene was inserted in the highly variable region of nsp2. The V132A and P134A mutations were introduced into the nsp1α-coding region to create the mutant full-length cDNA clones. **B** Recovery of recombinant viruses through DNA transfection of full-length cDNA clones. BHK-21 cells were transfected with the WT full-length cDNA clone or its mutants (V132A and P134A). At 48 hpt, the transfection efficiency was determined by the expression of EGFP (green fluorescence), and culture supernatants were harvested to infect MARC-145 cells. Nuclei were counterstained with DAPI. The scale bar is 100 μm. The expression of nsp2 and N in BHK-21 cells was also evaluated by western blot analysis (**C**). β-tubulin was detected as a loading control. **D** Infection of the recombinant viruses in MARC-145 cells. MARC-145 cells were inoculated with culture supernatants harvested from BHK-21 cells by transfection. The EGFP signal and cytopathic effect (CPE) were monitored daily to determine the viability of the recombinant viruses. The scale bar is 100 μm. **E** Verification of vV132A by genomic sequencing. The nsp1α-coding region was determined by RT-PCR and DNA sequencing for passage three WT and vV132A.

**Figure 6:**
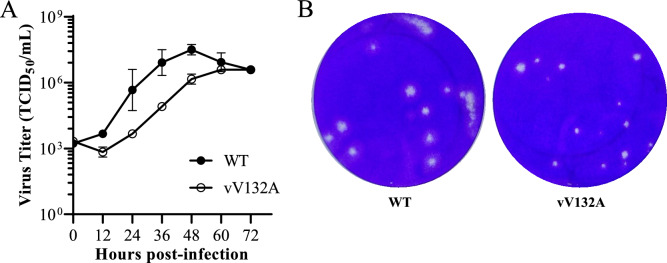
**In vitro characterization of the recombinant viruses.** **A** Growth kinetics of WT and vV132A MARC-145 cells. MARC-145 cells were infected with the indicated viruses at an MOI of 0.01. At the indicated time points, culture supernatants were harvested and subjected to viral titration in MARC-145 cells. The data points represent the mean ± SD. **B** Plaque morphology of WT and vV132A MARC-145 cells. Confluent cell monolayers were infected with two-fold serially diluted virus. The cell monolayers overlaid with 1% low-melting agar in MEM supplemented with 2% FBS were maintained in a cell culture incubator. At 5 dpi, the cell monolayers were fixed with 4% paraformaldehyde, followed by staining with 0.1% crystal violet to visualize the plaques.

To further investigate the effect of nsp1α dimerization on PRRSV replication, the V132A and P134A mutations in nsp1α were introduced into the full-length cDNA clone (pCMV-TA-EGFP) of the TA-12 strain (Figure 5A). BHK-21 cells were transfected with cDNA clones to rescue recombinant viruses. Similar transfection efficiencies of all three cDNA clones were demonstrated by similar expression levels of nsp2-EGFP (Figures 5B and C). The N protein was detected in cells transfected with the WT or the V132A mutant but not in those transfected with the P134A mutant (Figure 5C). The culture supernatants were collected to inoculate MARC-145 cells. As indicated by the expression of nsp2-EGFP, we successfully recovered the V132A mutant (vV132A) but not the P134A mutant (Figure 5D). The vV132A mutation was confirmed by DNA sequencing of the nsp1α-coding region (Figure 5E). The recombinant viruses were serially passaged in MARC-145 cells. The V132A mutant remained genetically stable according to DNA sequencing of the passage 5 and passage 10 viruses. Compared with the WT virus, which peaked titre of 10^7.5^ TCID_50_/mL, vV132A presented significantly impaired replication capacity, resulting in a tenfold reduction in the peak viral titre and consistently delayed viral kinetics throughout the time course (Figure 6A). Consistently, vV132A infection generated smaller plaques than did the WT virus (Figure 6B). The observation that the P134A mutation disrupts nsp1α dimerization and completely abolishes virus recovery strongly indicates that the dimeric conformation of nsp1α is indispensable for PRRSV viability.

### The dimeric conformation of nsp1α plays a key role in regulating viral RNA synthesis

**Figure 7:**
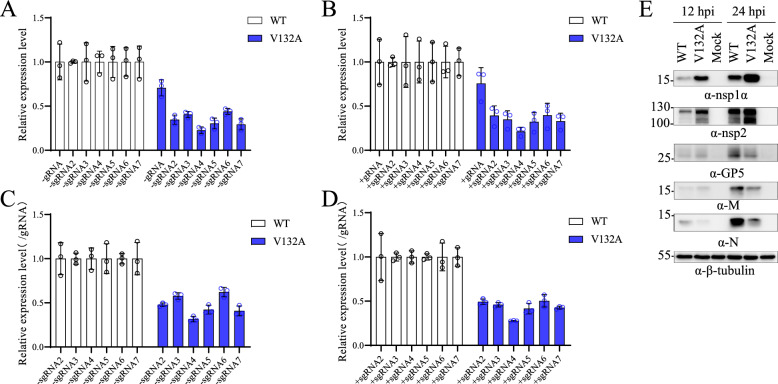
**The V132 residue is important for the regulation of the relative accumulation of subgenomic RNAs by nsp1α**. **A** The expression levels of negative-strand viral RNAs. **B** The expression levels of positive-strand viral RNAs. **C** The relative expression levels of negative-strand viral subgenomic RNAs compared with those of negative-strand viral genomic RNA. **D** The relative expression levels of subgenomic mRNAs compared with those of viral genomic RNA. MARC-145 cells were infected with WT or vV132A (passage 3) at an MOI of 3. At 9 hpi, the cells were harvested for total cellular RNA extraction. The accumulation of viral RNAs, including positive-strand genomic RNA (+gRNA) and subgenomic RNAs (+sgRNA) and negative-strand genomic RNA (−gRNA) and subgenomic RNAs (−sgRNA), was quantified by RT‒qPCR with the primers listed in Table 2. The expression of viral RNA was normalized to the expression of the housekeeping gene β-actin. The relative expression levels of both positive- and negative-sense subgenomic RNAs were calculated by normalizing their expression values to those of genomic RNA. **E** The expression of selected viral proteins in MARC-145 cells infected with WT or vV132A. MARC-145 cells were infected at an MOI of 3. At 12 hpi and 24 hpi, the cell lysates were harvested and subjected to western blot detection of viral proteins, including nsp1α, nsp2, GP5, M, and N, while β-tubulin was detected as a loading control.

To further study the role of nsp1α dimerization in viral replication, a panel of primers was designed for RT‒qPCR to quantify the synthesis of viral RNA species (Additional file 1A). The specificity of these primers was initially verified by RT‒PCR (Additional file 1B). In MARC-145 cells infected with vTA-EGFP (WT) or vV132A at an MOI of 3, the expression levels of viral RNAs were normalized to those of host β-actin mRNA at 9 hpi. Compared with the WT virus, vV132A produced much lower levels of viral RNAs, especially the subgenomic RNAs of both senses (Figures 7A and B). In EAV, the optimal ratios between viral genomic and subgenomic RNAs (sgRNA) are critical for efficient viral replication, and nsp1 modulates the accumulation of minus-strand RNAs [21]. To investigate the potential differential regulatory role of nsp1α in viral RNA synthesis, we quantified the relative expression levels of both positive- and negative-sense subgenomic RNAs by normalizing their expression values to those of genomic RNA. As shown in Figures 7C and D, the relative expression levels of subgenomic RNAs were significantly lower than those of the WT virus. We further compared the expression levels of viral nonstructural proteins (nsp1α and nsp2) and structural proteins (GP5, M, and N) between the WT virus and vV132A at 12 hpi and 24 hpi. Compared with the WT virus, vV132A resulted in increased expression levels of nonstructural proteins but decreased expression levels of structural proteins (Figure 7E), which is consistent with the reduced relative levels of subgenomic RNAs (Figures 7C and D). These findings reveal that the V132 residue is involved in differentially regulating viral RNA synthesis.

**Figure 8:**
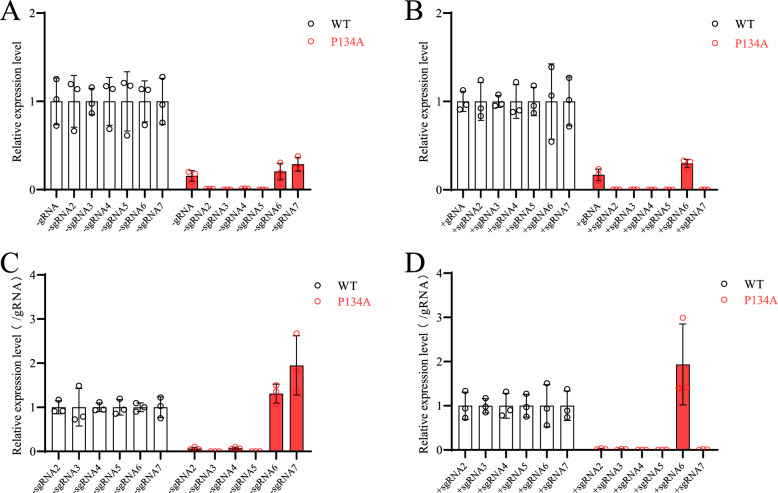
**The P134 residue is essential for the synthesis of several subgenomic RNAs**. **A** The expression levels of negative-strand viral RNAs. **B** The expression levels of positive-strand viral RNAs. **C** The relative expression levels of negative-strand viral subgenomic RNAs compared with those of negative-strand viral genomic RNA. **D** The relative expression levels of subgenomic mRNAs compared with those of viral genomic RNA. BHK-21 cells were transfected with the WT full-length cDNA clone or the P134A mutant. At 24 hpt, the cells were harvested for total cellular RNA extraction, and DNA contamination was removed by DNase I treatment. The accumulation of viral RNAs, including positive-strand genomic RNA (+gRNA) and subgenomic RNAs (+sgRNA) and negative-strand genomic RNA (-gRNA) and subgenomic RNAs (−sgRNA), was quantified by RT‒qPCR with the primers listed in Table 2. The expression of viral RNA was normalized to the expression of the housekeeping gene β-actin. The relative expression levels of both positive- and negative-sense subgenomic RNAs were calculated by normalizing their expression values to those of genomic RNA.

Since the P134 residue is essential for nsp1α dimerization, we further investigated viral RNA synthesis in BHK-21 cells transfected with the full-length cDNA clone of the P134A mutant. For negative-sense viral RNAs, sgRNAs 2 ~ 5 of the P134A mutant were undetectable, whereas the gRNA and sgRNAs 6 ~ 7 of the P134A mutant were significantly downregulated compared with those of the WT virus (Figure 8A). For positive-sense viral RNAs, only gRNA and sgRNA6 were detected for the P134A mutant, and their expression levels were also significantly lower than those of the WT virus (Figure 8B). On the basis of the relative expression levels of the sgRNAs, we found that nsp1α-P134A did not affect the synthesis of the negative-sense sgRNA6 and sgRNA7 or the positive-sense sgRNA6 and almost completely blocked the other subgenomic RNAs (Figure 8C and D). These results suggest that the P134 residue of nsp1α is essential for the synthesis of negative-sense sgRNAs 2–5 and positive-sense sgRNA7.

In summary, these findings reveal that the dimeric conformation of nsp1α is essential for its ability to facilitate the synthesis of PRRSV subgenomic RNAs.

## Discussion

As the first viral protein translated during PRRSV infection, nsp1α plays various roles in viral RNA synthesis and virus‒host interactions [9, 12, 17]. The crystal structure of nsp1α, purified from *E. coli*, reveals a stable dimeric conformation in solution, with a distinct set of residues identified as potential mediators of this self-interaction [15]. This dimerization has been further confirmed by Co-IP analysis in overexpression systems [16]. Notably, nsp1α is not unique in its oligomerization, as several other PRRSV proteins, including nsp9, nsp12, and the N protein, form functionally significant homodimers [14, 16, 33]. To date, the biological relevance of nsp1α homodimerization during PRRSV infection remains elusive, particularly regarding its functional implications and potential role in viral replication or host immune modulation. To address this knowledge gap, we systematically investigated the functional significance of the nsp1α dimeric conformation using multiple experimental systems.

Structural analysis of PRRSV nsp1α revealed that multiple hydrophobic residues contribute to self-interaction, as illustrated in Figure 1A [15]. Through comprehensive mutagenesis studies, we identified residues at positions 132 and 134 as the key residues for nsp1α dimerization across both PRRSV species. GST pulldown assays demonstrated that both mutants exhibited diminished self-interaction capabilities, with the V132A mutant retaining relatively stronger self-interactions than the P134A mutant did. Notably, while position 134 is completely conserved across all PRRSV strains, position 132 shows distinct but highly conserved residues between PRRSV-1 and PRRSV-2. Interestingly, interchanging the 132nd residue between PRRSV-1 and PRRSV-2 did not disrupt nsp1α dimerization. To investigate nsp1α dimerization during viral infection, we generated recombinant viruses expressing FLAG-tagged nsp1α, enabling the detection of self-interaction. Both colocalization and Co-IP analyses confirmed the self-interaction of nsp1α during infection, with positions 132 and 134 proving essential for dimerization. Furthermore, the V132A mutant retains partial self-interactivity, as evidenced by the observed colocalization between nsp1α and FLAG-nsp1α-V132A. These findings reveal the critical role of residues at positions 132 and 134 in the dimeric conformation of nsp1α.

PCPα activity of PRRSV nsp1α is critical for its release from the polyprotein pp1a. Owing to their location on opposite sites of each nsp1α homodimer, the two protease active sites could function independently [15]. Here, we tested the impact of the dimeric conformation on PCPα activity using the dimerization-deficient mutants (V132A and P134A). As expected, the autoproteolytic activity of nsp1 was not affected by these mutations. As a major immune antagonist, nsp1α employs multiple strategies to evade host innate and cell-mediated immunity [23, 24, 26–29, 31]. We tested whether dimerization-deficient mutations affect the inhibitory effect of nsp1α on type I IFN production and the degradation of SLA-I molecules. The inhibitory effect of nsp1α on type I IFN production was significantly reduced by the N91A and N161A mutations, which are associated with its ability to degrade SLA-I molecules, but was not affected by the mutations disrupting its dimeric conformation. Compared with WT nsp1α, the dimerization-deficient mutants also demonstrated similar ability to degrade SLA-I molecules. Thus, nsp1α dimerization does not appear to directly affect its immune antagonist activity. The observation that dimerization-deficient mutants retain both protease activity and immune antagonist functionality strongly suggests that the overall structural integrity of nsp1α is largely preserved despite these specific mutations.

*Arteriviruses* employ a discontinuous negative-strand RNA synthesis mechanism to transcribe a 3’-co-terminal nested set of sg mRNAs that encode viral structural proteins. Each sg mRNA shares a common 5’ leader sequence identical to the 5’UTR of the genome [34]. This process depends on short conserved transcription regulatory sequences (TRSs) and replication–transcription complexes formed by nonstructural proteins, as demonstrated in EAV and PRRSV using reverse genetics [35–37]. In EAV, specific mutations in nsp1 and nsp10 can disrupt sg mRNA synthesis [19, 38], with mutations in the nsp1 N-terminal zinc finger domain affecting the balanced accumulation of genomic and subgenomic mRNAs by altering negative-strand template levels [21]. In addition to the N-terminal ZF domain, other nsp1 domains also play roles in this process [19]. In PRRSV, the nsp1α and nsp12 subunits appear to facilitate the synthesis of all negative-strand sg RNAs during transcriptional regulation [14, 17], with nsp12 requiring a covalent disulfide bond-mediated dimeric conformation for this activity [14]. Our investigation of the role of nsp1α in transcriptional regulation using an HP-PRRSV replicon system revealed that V132A and P134 mutations differentially affected Gluc expression, increasing the expression of nonstructural proteins (nsp1α and nsp2) but not structural proteins (N), suggesting a disrupted balance in genomic and subgenomic RNA accumulation. Compared with wild-type infection, the V132A mutation consistently reduced the relative accumulation of all viral subgenomic RNAs, with protein expression patterns strongly correlated with mRNA levels. The P134A mutation, which prevented recombinant virus rescue, was characterized through DNA transfection of BHK-21 cells, revealing near-complete blockage of negative-strand sgRNA 2–5 and positive-strand sgRNA7 synthesis, along with reduced genome-length RNA and sgRNA6 levels. Interestingly, the relative accumulation of sgRNA6 in both senses was not affected. These findings suggest that the dimeric conformation of nsp1α plays a crucial role in supporting subgenomic RNA synthesis at both the negative- and positive-strand levels. Furthermore, the relative accumulation of subgenomic RNAs could be differentially regulated by dimerization-deficient nsp1α mutants.

In summary, this study identified V132 and P134 as critical residues governing the dimeric conformation of nsp1α, with the V132A mutation maintaining partial self-interactivity and the P134A mutation completely disrupting dimerization. Our findings reveal a functional dichotomy in nsp1α activities: while its dimerization is essential for viral RNA synthesis and transcriptional regulation, it appears to be dispensable for both the autoproteolytic activity and immune evasion functions of PCPα. The distinct impacts of the V132A and P134 mutations on subgenomic RNA production underscore the sophisticated regulatory role of nsp1α dimerization in maintaining the precise balance between genomic and subgenomic RNA synthesis. These findings significantly advance our understanding of the molecular mechanisms of nsp1α and provide novel insights into arterivirus replication and transcriptional regulation. The identification of these key dimerization residues not only enhances our fundamental knowledge of PRRSV biology but also provides a foundation for the development of targeted antiviral strategies against PRRSV and related arteriviruses.

## Supplementary Information

**Additional file 1.** **Strategy for RT‒qPCR quantification of PRRSV RNA. A** The sites on viral RNAs targeted by primers for RT‒qPCR detection. The primers for genomic RNA detection anchor the nsp1-coding region, whereas the primers for subgenomic RNA detection target the leader–body junction sites. **B**. The detection specificity of the primers for RT‒qPCR was verified by RT‒PCR with total RNA from MARC-145 cells with or without PRRSV infection.

## Data Availability

All the data generated or analysed in this study are included in the manuscript and its supplementary information file.
