# Supplementary material for: The dimeric conformation of PRRSV nsp1α is important for its ability to regulate viral RNA synthesis
Source: Vet Res. 2025 May 21;56:105. doi: 10.1186/s13567-025-01537-5 (PMC12096626; doi:10.1186/s13567-025-01537-5)
Supplement: Supplementary file 1 — Additional file 1. Strategy for RT‒qPCR quantification of PRRSV RNA. A The sites on viral RNAs targeted by primers for RT‒qPCR detection. The primers for genomic RNA detection anchor the nsp1-coding region, whereas the primers for subgenomic RNA detection target the leader–body junction sites. B. The detection specificity of the primers for RT‒qPCR was verified by RT‒PCR with total RNA from MARC-145 cells with or without PRRSV infection. [file 13567_2025_1537_MOESM1_ESM.pptx]

## Slide 1
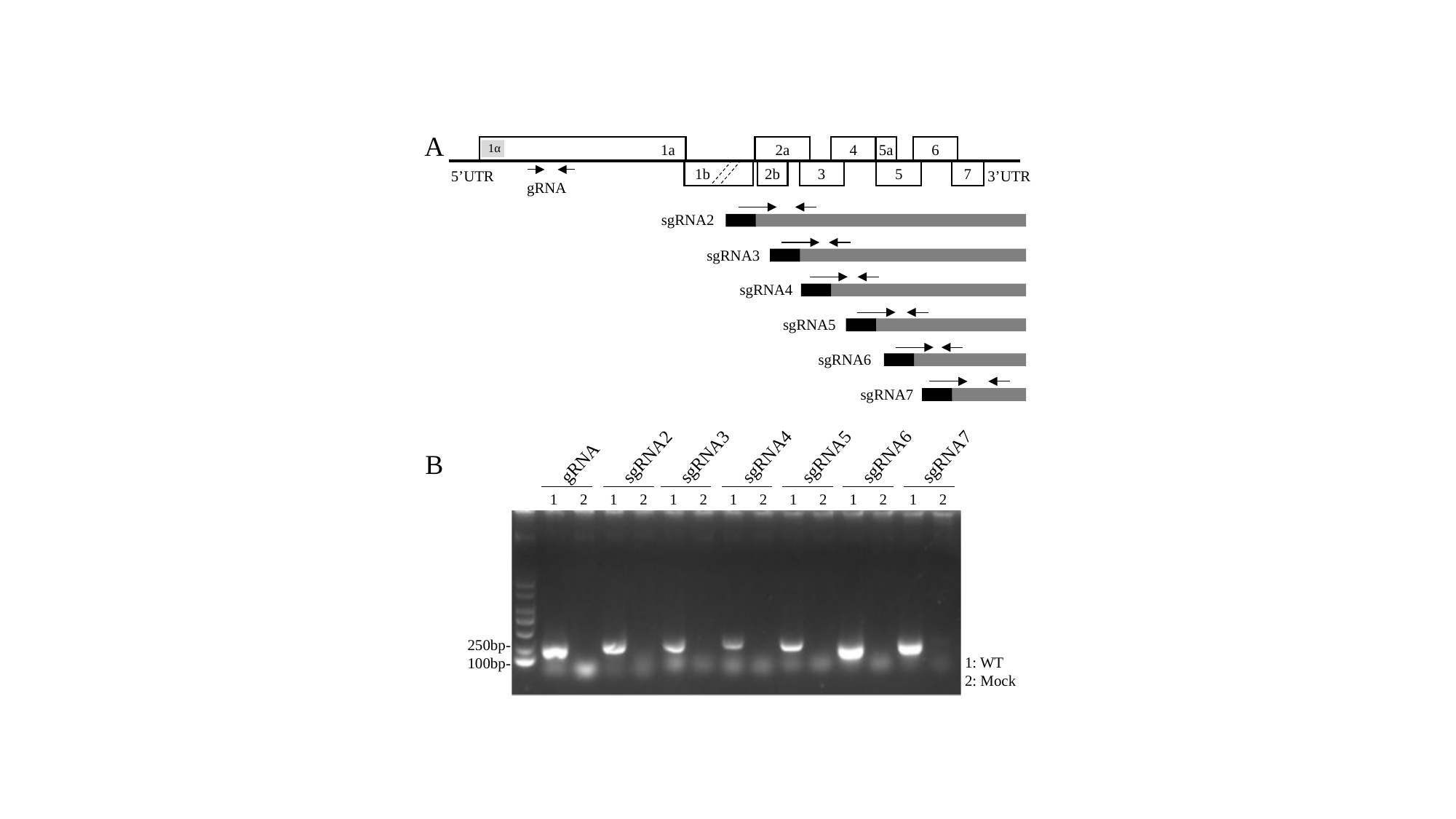

A
1α
5a
1a
2a
4
6
5’UTR
3’UTR
1b
2b
3
5
7
gRNA
sgRNA2
sgRNA3
sgRNA4
sgRNA5
sgRNA6
sgRNA7
sgRNA2
sgRNA3
sgRNA4
sgRNA5
sgRNA6
sgRNA7
gRNA
1
2
1
2
1
2
1
2
1
2
1
2
1
2
250bp-
1: WT
2: Mock
100bp-
B
